# Nail-preserving excision of glomus tumor in the second toe: Case report and literature review

**DOI:** 10.1097/MD.0000000000037398

**Published:** 2024-03-15

**Authors:** Young Uk Park, Jongseong Han, Young Wook Seo

**Affiliations:** a Department of Orthopedic Surgery, Ajou University Hospital, Ajou University School of Medicine, Suwon, Gyeonggi-do, Korea.

**Keywords:** color duplex ultrasound, glomus tumor, toe, transungal approach

## Abstract

**Introduction:**

This case report describes the diagnosis of a glomus tumor in the second toe of a 38-year-old female, followed by surgical treatment utilizing a transungual approach to preserve the nail. This study highlights the diagnostic challenges and surgical strategies to treat such tumors while preserving nail integrity.

**Patient concerns:**

Pain occurred once a week, but over time, it increased, and just before seeking medical attention, she experienced pain more than 5 times a day. The pain worsened when cold water touched her toe.

**Diagnosis:**

We observed a slight hump indicating nail plate deformity, and the patient exhibited severe pinpoint tenderness (positive Love test) in the affected area. Color duplex ultrasound was performed for further investigation, revealing a hypervascular hypoechoic nodule measuring 0.5 cm in size at the nail bed of the right second toe.

**Intervention:**

The surgery was performed under digital nerve block anesthesia using a modified transungual nail-preserving approach for the excision of the glomus tumor.

**Outcomes:**

The pain that was reported prior to the surgery has improved postoperatively, and the recovery has been uneventful without any other complication.

**Conclusion:**

This paper provides a comprehensive examination of a rare glomus tumor in the second toe, elucidating both diagnostic intricacies and treatment modalities. It emphasizes the dual necessity of achieving total tumor excision while also considering aesthetic outcomes. The insights presented herein are intended to serve as valuable guidance for clinicians confronted with similar clinical scenarios, underlining the delicate interplay between effective tumor management and the preservation of cosmetic integrity.

## 1. Introduction

Glomus tumor is a benign neoplasm that originates from the glomus body, which is a contractile neuromyoarterial structure capable of regulating peripheral blood flow, blood pressure, and temperature.^[1]^ In the majority of cases, glomus tumors manifest as solitary lesions, typically measuring <1 cm in size.^[2]^ These tumors are commonly observed in individuals aged between 30 and 50 years old.^[3]^ Glomus tumors are considered rare, accounting for approximately 1% to 5% of all tumors in the lower extremities,^[4]^ and comprising <2% of soft tissue tumors.^[5,6]^ Anatomically, glomus tumors tend to occur with a higher density of glomus bodies in the subungual zone of the fingertip.^[7]^ Approximately 75% of glomus tumors occur in the hands, particularly in the subungual region. The remaining cases are observed in other areas, such as the feet. Extradigital lesions are rare, but they have been reported in various locations, including the head, neck, stomach, lung, tongue, colon, bladder, and coccyx.^[6,8]^ For glomus tumors in the extremities, it has been reported that the average time from symptom onset to accurate diagnosis is around 10 years, and patients typically consulted an average of 2.5 physicians before receiving the correct diagnosis.^[9]^ The main triad of symptoms includes excruciating pain in the affected finger, cold intolerance, and typical discoloration.^[10]^ Glomus tumors can induce severe tenderness and often lead to nail deformity. Although complete surgical resection is recommended as the treatment method, it is challenging due to their frequent occurrence in the subungual region, and it is prone to nail deformity and tumor recurrence.^[11,12]^ Various surgical approaches, including the transungual and periungual techniques, have been described by several authors in the medical literature.^[13]^

In this case presentation, we opted for a technique that preserves the nail while adequately exposing the nail bed to achieve complete tumor removal. This paper aims to share the course of an uncommon glomus tumor in the second toe, with the purpose of providing insights into diagnosis and treatment, hoping to be helpful in clinical practice.

## 2. Case report

The patient in this case is a 38-year-old female with no significant medical history except for bilateral polycystic kidneys. She did not experience any foot pain before, but 4 months ago, she began to feel pain in her right second toe. Initially, the pain occurred once a week, but over time, it increased, and just before seeking medical attention, she experienced pain more than 5 times a day. The pain worsened when cold water touched her toe. Wearing shoes also caused severe pain. After undergoing magnetic resonance imaging (MRI) at other hospitals’ orthopedic and dermatology departments, the patient was referred to a tertiary care hospital, where she presented at our outpatient clinic.

**Figure 1.**
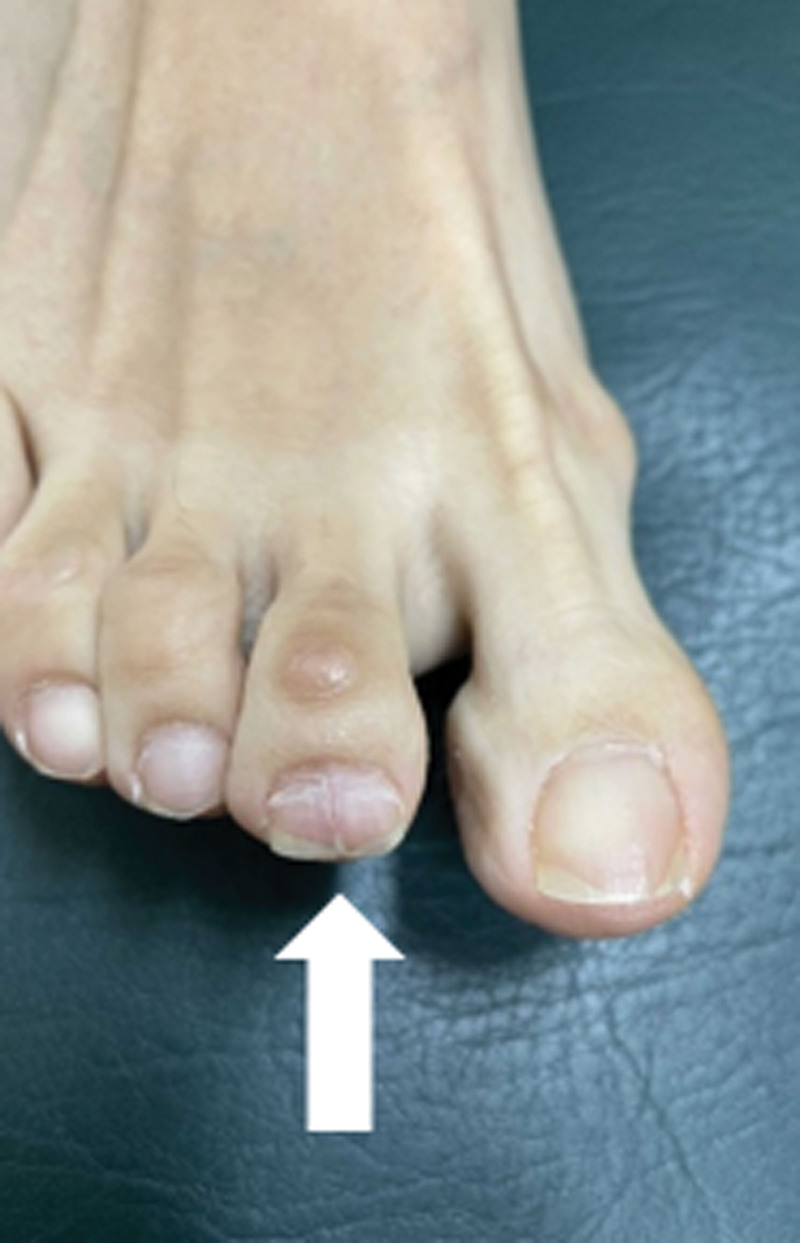
Photograph showing subungual glomus tumor at the right second toe, marked with arrows. Note the slight irregular hump deforming the nail plate into a convex shape.

During the physical examination conducted at hospital, we observed a slight hump indicating nail plate deformity, and the patient exhibited severe pinpoint tenderness (positive Love test) in the affected area (Fig. 1).

**Figure 2.**
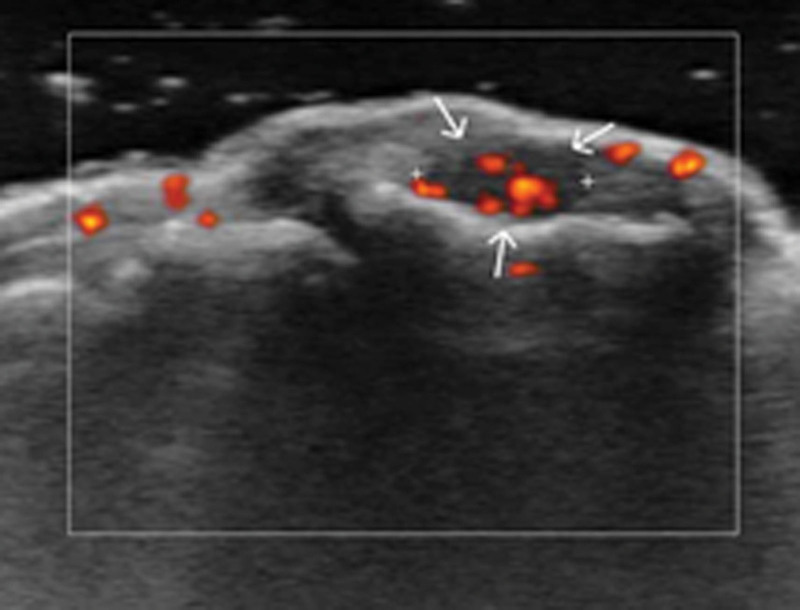
0.5 cm sized hypervascular hypoechoic nodule at the nail bed of the right second toe.

**Figure 3.**
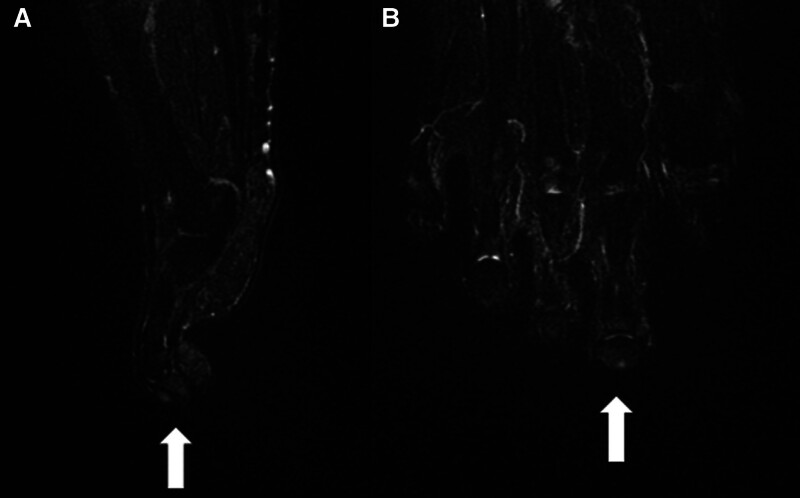
0.4 cm sized focal soft tissue mass showing high signal intensity in T2 (arrow).

The X-ray conducted on the same day of presentation did not reveal any significant bony abnormalities. Color duplex ultrasound was performed for further investigation, revealing a hypervascular hypoechoic nodule measuring 0.5 cm in size at the nail bed of the right second toe (Fig. 2). An MRI conducted at another hospital 4 months prior to the current visit revealed a focal soft tissue mass measuring 0.4 cm in size, displaying high signal intensity on the T2-weighted image (Fig. 3).

**Figure 4.**
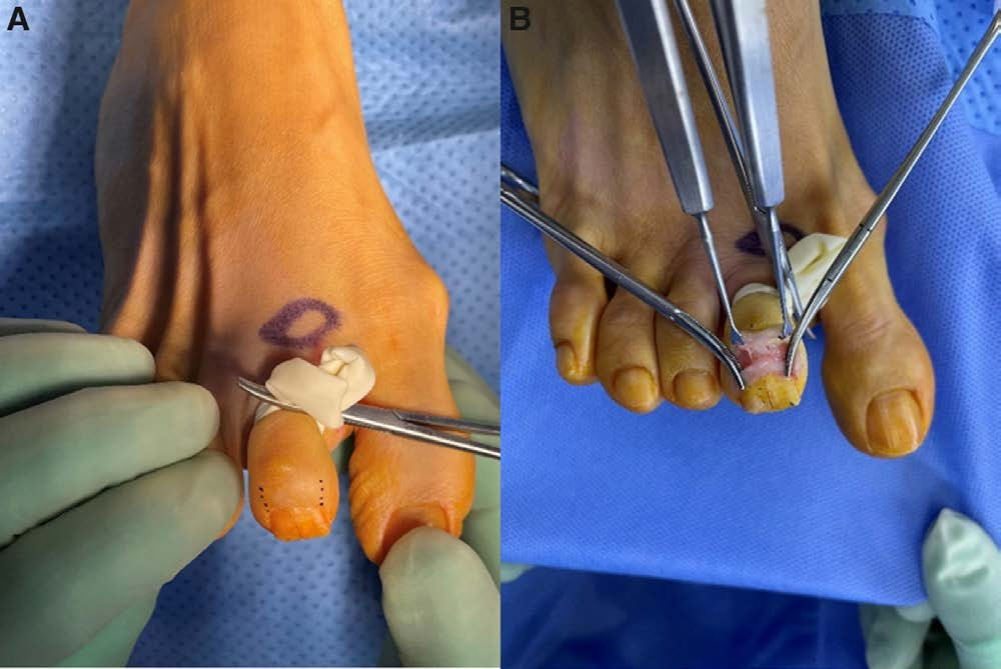
The surgery was performed under digital nerve block anesthesia using a transungual nail-preserving approach for the excision of the glomus tumor. (A) Markings were made prior to the start of the surgery. (B) Nail bed following the incision.

**Figure 5.**
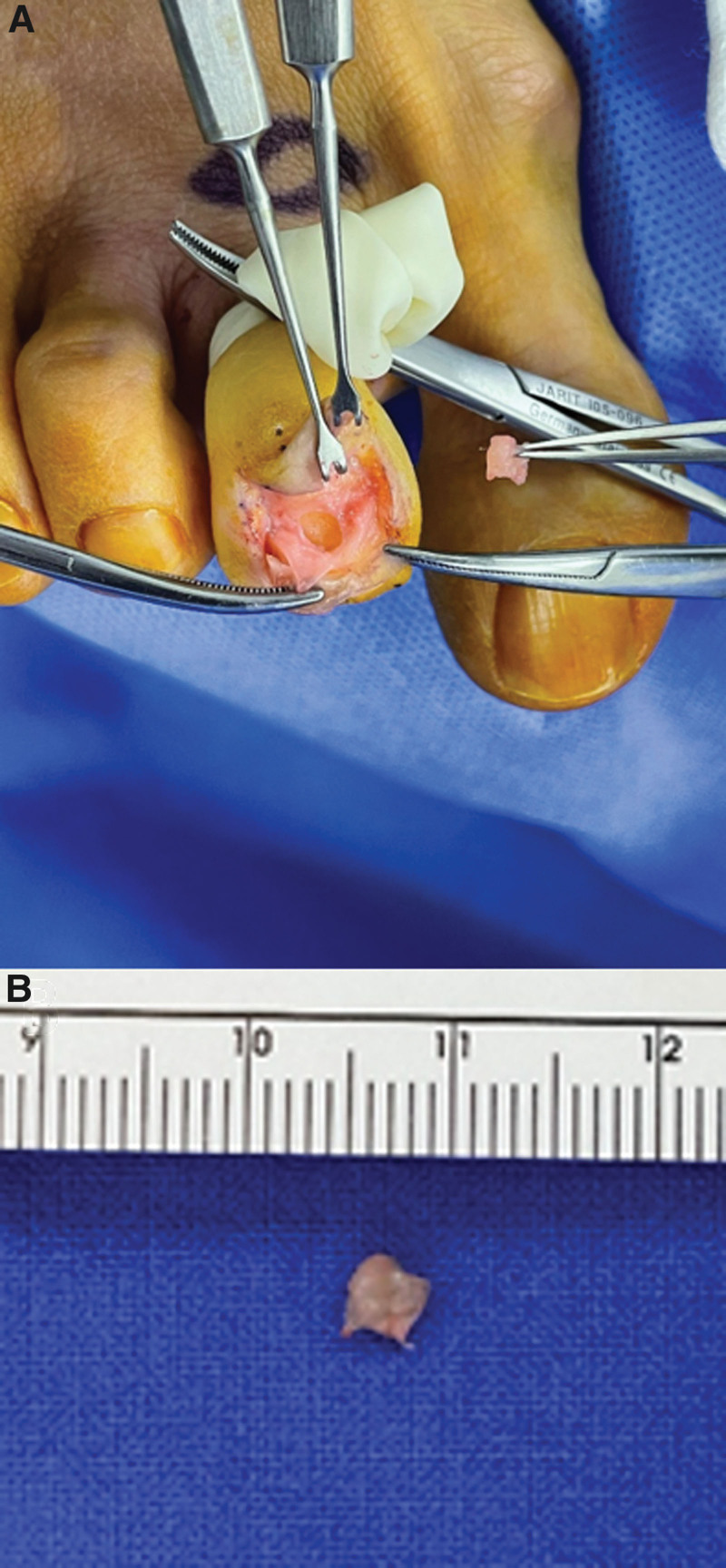
(A) The nail bed was longitudinally incised to visualize the glomus tumor. (B) The glomus tumor after its surgical removal.

**Figure 6.**
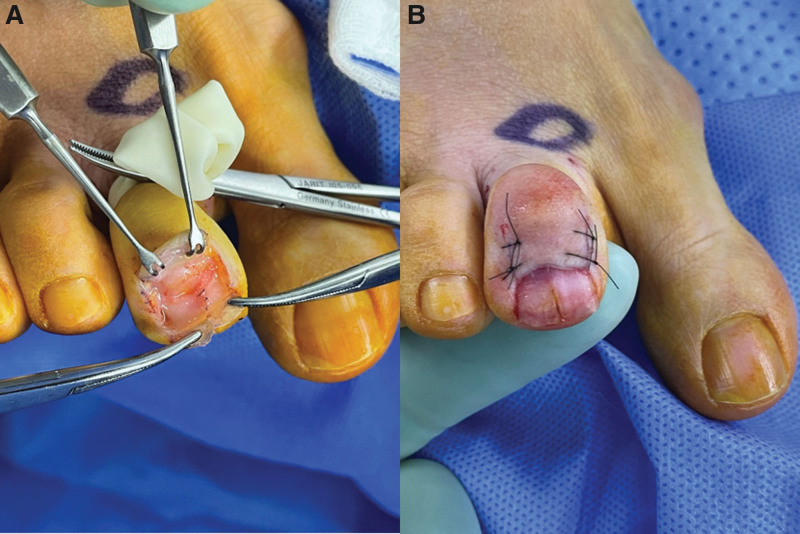
(A) After excising the tumor, the nail bed was repaired using vicryl 6-0. (B) The elevated nail was repositioned by reducing it and the nail fold was repaired. A simple suture was performed using nylon 5-0.

The surgery was performed under digital nerve block anesthesia using a modified transungual nail-preserving approach for the excision of the glomus tumor. A tourniquet was applied to the second toe, and the area of the skin incision was marked (Fig. 4A). After making an approximately 3 cm skin incision in a U-shaped fashion around the proximal base of the second toe nail, the nail fold area is dissected, and the tissue is lifted in the proximal direction. The proximal nail fold was lifted inward using a skin hook, and the proximal end of the nail plate was gently raised in a distal direction to expose the nail bed (Fig. 4B). A comprehensive examination was conducted on the exposed nail bed and its surrounding matrix. A longitudinal incision was subsequently made in the nail bed, with preservation of the distal portion of the nail (Fig. 5A). A white-pinkish encapsulated mass with a diameter of 5 mm was excised, and there were no signs of invasion into the surrounding soft tissue (Fig. 5B) After excising the tumor, the nail bed was repaired using vicryl 6-0 (Fig. 6A). The elevated nail was repositioned by reducing it and the nail fold was repaired. A simple suture was performed using nylon 5-0 (Fig. 6B).

**Figure 7.**
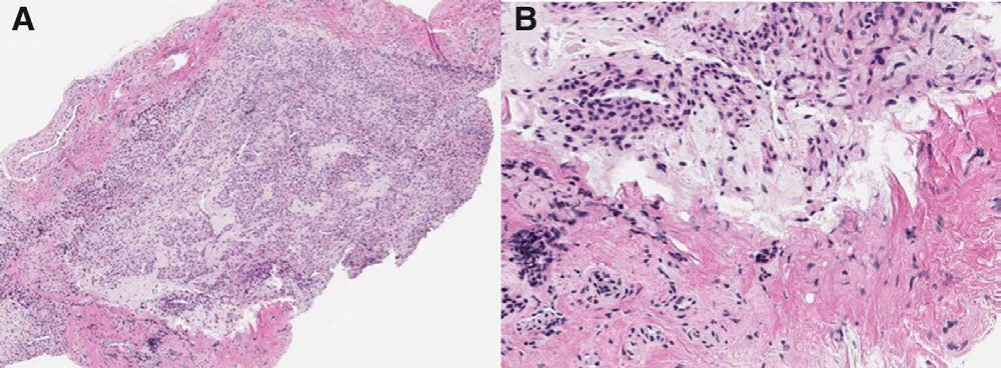
Histopathological studies. (A) In the histopathological analysis, under low-power magnification, a well-circumscribed and encapsulated tumor composed of a proliferation of tumor cells in a myxoid stroma was observed. (B) Under high-power magnification, the tumor cells appeared polygonal and had round nuclei with smooth nuclear contours, along with a moderate amount of eosinophilic cytoplasm.

**Figure 8.**
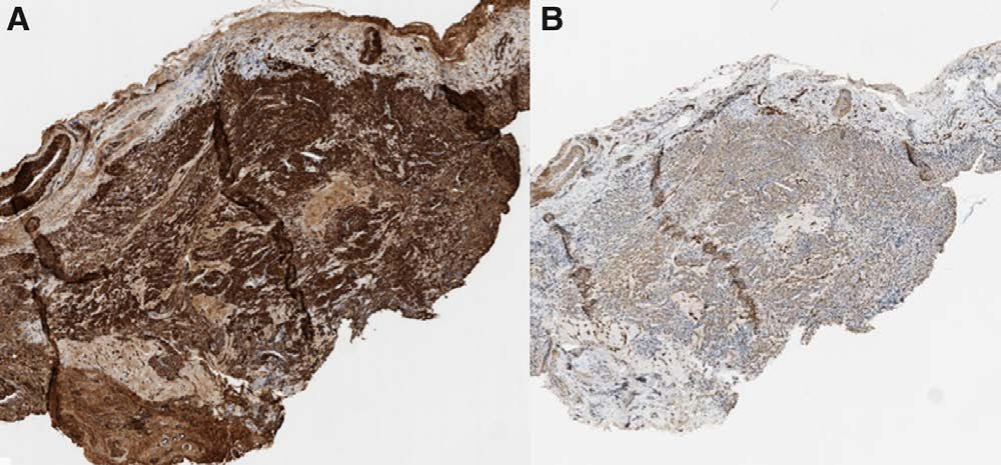
Immunohistochemical studies. (A) In the immunohistochemical study, the tumor showed positivity for SMA. (B) Weak positivity for S-100. SMA = smooth muscle actin.

Histopathological and immunohistochemical studies were conducted on the specimens obtained after the surgery. In the histopathological analysis, under low-power magnification, a well-circumscribed and encapsulated tumor composed of a proliferation of tumor cells in a myxoid stroma was observed (Fig. 7A). Under high-power magnification, the tumor cells appeared polygonal and had round nuclei with smooth nuclear contours, along with a moderate amount of eosinophilic cytoplasm (Fig. 7B). These findings are consistent with those of a glomus tumor. In the immunohistochemical study, the tumor showed positivity for smooth muscle actin (Fig. 8A) and weak positivity for S-100 (Fig. 8B). These findings supported the diagnosis of a glomus tumor.

**Figure 9.**
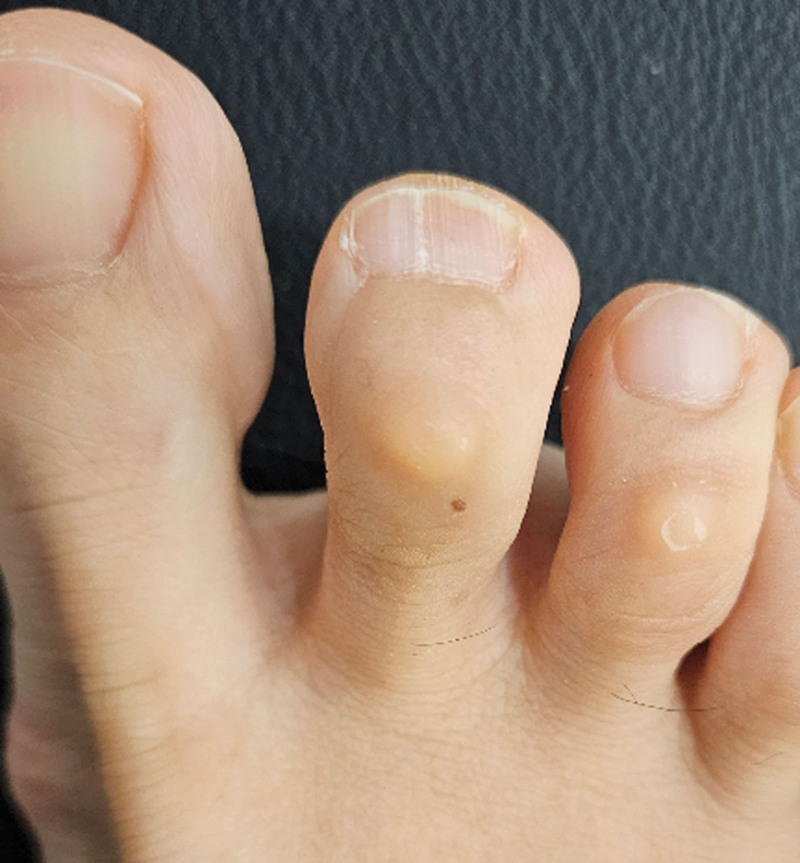
Clinical photo of the right second toe at 24 mo post-surgery.

The patient has been under outpatient follow-up for 24 months after the surgery. All previously reported symptoms, including pain, had improved. The pain score improved from 5 to 6 on the visual analog scale preoperatively to 0 postoperatively. There was no evidence of a wound scar, and the nail was successfully restored and maintained (Fig. 9). Furthermore, there were no signs of recurrence.

## 3. Discussion

The glomus tumor was first described by Barre and Masson in 1924.^[14]^ Glomus tumor is a rare benign neoplasm that arises from the glomus body, which is involved in temperature regulation and blood flow.

Glomus tumor accounts for 1% to 5% of tumors in the subungual region and <2% of tumors in soft tissues.^[4–6]^ According to Gombos et al,^[15]^ glomus tumors occurring in the foot are relatively rare compared to their incidence in the subungual region, which can often result in delays in both diagnosis and treatment. While the majority of glomus tumors are benign, around 1% are identified as malignant.^[3]^ Tumors larger than 2 cm in size, located deeply, and showing the presence of atypical mitotic figures may display malignant histopathological features. If malignancy is identified through histological examination, the likelihood of metastasis can be considered to be higher than 25%. Histopathological examination is essential to exclude the presence of malignancy.^[16]^

The classic triad of clinical symptoms consists of aching pain, exquisite focal tenderness, and cold hypersensitivity.^[1]^ A variety of clinical diagnostic tests can improve the accuracy of the diagnosis. The Love test, which involves confirming pin-point tenderness, is available (sensitivity: 100% and specificity: 78%). Additionally, the Hildreth test, which observes the disappearance of pain when the tourniquet is applied, is present (sensitivity: 71.4%, specificity: 100%). Furthermore, the cold sensitivity test, which demonstrates worsened pain with cold water and alleviation with heat, is also available (with the highest specificity, sensitivity, and accuracy at around 100%). The combination of these 3 tests can elevate the precision of diagnosis.^[17]^

X-ray imaging often reveals phalangeal bony erosion; however, it is not suitable as a discriminatory diagnostic test.^[18]^ Color duplex ultrasound is suitable for diagnosing masses smaller than 2 mm, with a specificity of 67% and a detection rate of 100% without false negatives.^[19,20]^ On ultrasound examination, it typically presents a characteristic hypervascular hypoechoic mass appearance. MRI is also valuable for detecting small masses of about 2 mm in size, showing low intensity on T1 and hyperintensity on T2. However, it has a negative predictive value of 20% and specificity of 50% compared to its high sensitivity of 90% and positive predictive value of 97%, rendering it less cost-effective and practical compared to ultrasound.^[21]^

Treatment typically involves surgical intervention, although due to its location within the nail matrix, complete excision can be challenging, leading to a tendency for recurrence.^[11]^ Expanding the surgical field for complete removal can increase the risk of damaging the nail matrix. Consequently, surgeons must carefully consider their approach.^[22]^ Preserving the nail bed during the removal of a glomus tumor is crucial. Partial or complete removal of the nail plate provides good exposure but can lead to a prolonged period for normal nail regrowth and the potential for damage to the nail bed. On the other hand, many authors have shown favorable outcomes with a transungual approach.^[23,24]^

Despite its limitations, including a small sample size and its nature as an observational case report, this study offers valuable insights into a rare glomus tumor in the second toe. This study offers an in-depth exploration of a rare glomus tumor located in the second toe, shedding light on both the complex aspects of its diagnosis and the various treatment approaches. It holds significance for employing nail-preserving surgery to successfully remove the tumor.

## 4. Conclusion

This study provides a detailed investigation into a rare case of a glomus tumor in the second toe, emphasizing the diagnostic challenges and varied treatment options. Its uniqueness lies in addressing a subungual glomus tumor in this location and successfully implementing nail-preserving surgical techniques. This case contributes valuable knowledge to the field, especially in the surgical management of such rare tumors while maintaining aesthetic considerations.

## Author Contributions

**Conceptualization:** Young Wook Seo.

**Data curation:** Jongseong Han.

**Visualization:** Jongseong Han.

**Writing—original draft:** Young Uk Park.

**Writing—review & editing:** Young Wook Seo.

## References

[1] Shin DK Kim MS Kim SW. A painful glomus tumor on the pulp of the distal phalanx. J Korean Neurosurg Soc. 2010;48:185–7. 20856673 10.3340/jkns.2010.48.2.185 PMC2941867

[2] Radner H Blumcke I Reifenberger G. The new WHO classification of tumors of the nervous system 2000. Pathology and genetics. Pathologe. 2002;23:260–83. 12185780 10.1007/s00292-002-0530-8

[3] Tsuchie H Okada K Nagasawa H. Glomus tumor of the toe with symptoms similar to those of Morton’s disease. J Orthop Sci. 2009;14:826–9. 19997833 10.1007/s00776-009-1387-y

[4] Lui TH Mak SM. Glomus tumor of the great toe. J Foot Ankle Surg. 2014;53:360–3. 23860131 10.1053/j.jfas.2013.05.011

[5] Sprinkle RLB 3rd Sangueza OP Schwartz GA. Glomus tumor of the toe: an anatomical variant. J Am Podiatr Med Assoc. 2017;107:257–60. 28650755 10.7547/15-161

[6] Frikh R Alioua Z Harket A. Glomus tumors: anatomoclinical study of 14 cases with literature review. Ann Chir Plast Esthet. 2009;54:51–6. 18938010 10.1016/j.anplas.2008.05.001

[7] Jablon M Horowitz A Bernstein DA. Magnetic resonance imaging of a glomus tumor of the fingertip. J Hand Surg Am. 1990;15:507–9. 2161442 10.1016/0363-5023(90)90072-y

[8] Symmers WS. Glomus tumours. Br Med J. 1973;2:50–1. 10.1136/bmj.2.5857.50-b PMC1588991 4348689

[9] Van Geertruyden J Lorea P Goldschmidt D. Glomus tumours of the hand. A retrospective study of 51 cases. J Hand Surg Br. 1996;21:257–60. 8732413 10.1016/s0266-7681(96)80110-0

[10] Carroll RE Berman AT. Glomus tumors of the hand: review of the literature and report on twenty-eight cases. J Bone Joint Surg Am. 1972;54:691–703. 4341268

[11] Lin YC Hsiao PF Wu YH. Recurrent digital glomus tumor: analysis of 75 cases. Dermatol Surg. 2010;36:1396–400. 20629689 10.1111/j.1524-4725.2010.01647.x

[12] Tada H Hirayma T Takemitsu Y. Prevention of postoperative nail deformity after subungual glomus resection. J Hand Surg Am. 1994;19:500–3. 8056982 10.1016/0363-5023(94)90070-1

[13] Ekin A Ozkan M Kabaklioglu T. Subungual glomus tumours: a different approach to diagnosis and treatment. J Hand Surg Br. 1997;22:228–9. 9149994 10.1016/s0266-7681(97)80069-1

[14] Barre J Masson P. Anatomy–clinical study of certain painful subungual tumors (tumors of neuromyo-arterial glomus of the extremities). Bull Soc Dermatol Syph. 1924;31:148–59.

[15] Gombos Z Fogt F Zhang PJ. Intraosseous glomus tumor of the great toe: a case report with review of the literature. J Foot Ankle Surg. 2008;47:299–301. 18590892 10.1053/j.jfas.2008.04.003

[16] Polo C Borda D Poggio D. Glomus tumor of the hallux. Review of the literature and report of two cases. Foot Ankle Surg. 2012;18:89–93. 22443993 10.1016/j.fas.2011.05.005

[17] Bhaskaranand K Navadgi BC. Glomus tumour of the hand. J Hand Surg Br. 2002;27:229–31. 12074607 10.1054/jhsb.2001.0746

[18] Romanos E Al Delfi F Hubballah M. Glomus tumour of the fourth toe: case discussion and review of literature. BMJ Case Rep. 2019;12:e231100. 10.1136/bcr-2019-231100 PMC6887402 31772128

[19] Marchadier A Cohen M Legre R. Subungual glomus tumors of the fingers: ultrasound diagnosis. Chir Main. 2006;25:16–21. 16610516 10.1016/j.main.2005.12.007

[20] Chen SH Chen YL Cheng MH. The use of ultrasonography in preoperative localization of digital glomus tumors. Plast Reconstr Surg. 2003;112:115–9; discussion 120. 12832884 10.1097/01.PRS.0000066163.81401.05

[21] Al-Qattan MM Al-Namla A Al-Thunayan A. Magnetic resonance imaging in the diagnosis of glomus tumours of the hand. J Hand Surg Br. 2005;30:535–40. 16085343 10.1016/j.jhsb.2005.06.009

[22] Lee HJ Kim PT Kyung HS. Nail-preserving excision for subungual glomus tumour of the hand. J Plast Surg Hand Surg. 2014;48:201–4. 24256308 10.3109/2000656X.2013.861842

[23] Lee IJ Park DH Park MC. Subungual glomus tumours of the hand: diagnosis and outcome of the transungual approach. J Hand Surg Eur Vol. 2009;34:685–8. 19959449 10.1177/1753193408104799

[24] Jawalkar H Maryada VR Brahmajoshyula V. Subungual glomus tumors of the hand: treated by transungual excision. Indian J Orthop. 2015;49:403–7. 26229160 10.4103/0019-5413.159611 PMC4510793

